# A Case of Spinal Cord Stimulation Therapy for Lower Limb Pain Due to Conus Medullaris Syndrome

**DOI:** 10.7759/cureus.79469

**Published:** 2025-02-22

**Authors:** Yukari Yoshino, Tatsushige Iwamoto, Tomoyuki Matsumoto, Atsuhiro Kitaura, Yasufumi Nakajima

**Affiliations:** 1 Anesthesiology, Kindai University Faculty of Medicine, Osaka, JPN; 2 Anesthesiology and Critical Care, Kindai University Faculty of Medicine, Osaka, JPN; 3 Anesthesiology and Center for Outcomes Research, University of Texas Health Science Center, Houston, USA

**Keywords:** neuropathic pain, pain in the lower limbs, scs, spinal cord injury, upper spinal cone syndrome

## Abstract

This case report discusses the effective use of spinal cord stimulation (SCS) to treat lower limb pain caused by conus medullaris syndrome. A 49-year-old woman presented with persistent right lower limb pain and numbness following laparoscopic colon resection surgery. During epidural anesthesia, she experienced electric shock-like pain, which continued after surgery. MRI revealed fluid around the T12 thoracic spinal cord, suggesting spinal injury. Despite initial treatments, including epidural and nerve root blocks, the pain persisted. Further intervention with SCS led to significant pain reduction and improved mobility, enabling the patient to engage in rehabilitation.

Conus medullaris syndrome, typically involving the T11-T12 to L2 spinal cord levels, can cause varied symptoms, including muscle weakness, atrophy, and radicular pain. In this case, the injury at the T11-T12 level presented with pain localized to the L2-L3 nerve root area, complicating the diagnosis. SCS, known for its efficacy in managing neuropathic pain, proved to be a suitable treatment for this patient, whose pain was refractory to conservative therapies. The treatment led to sustained pain relief and facilitated rehabilitation.

This case highlights the diagnostic difficulties associated with conus medullaris syndrome; the symptoms of this condition may be similar to those of other conditions such as spinal pain. In this case, SCS was highly effective in treating neuropathic pain associated with spinal cord injury.

In conclusion, SCS therapy provides significant relief for patients with conus medullaris syndrome, underscoring its importance in the management of chronic intractable neuropathic pain.

## Introduction

It is estimated that approximately 500,000 spinal cord injuries (SCI) occur annually worldwide [1]. In this study, we presented a case of thoracolumbar transitional spinal cord injury after epidural anesthesia, followed by conus medullaris syndrome [2]. The diagnosis of conus medullaris syndrome is difficult because the symptoms may resemble nerve root or cauda equina lesions. This is most commonly L1, although the tip of the spinal conus terminates variably between thoracic body T11 and lumbar body L3. This diversity is due to the fact that the spinal cord and spinal column extend to different lengths during fetal development [3]. The spinal conus contains neural tissue from sacral medullary segments S2, S3, S4, S5, and caudal medullary segment 1. In the upper part of the body, the spinal conus regions are less well known because they extend from lumbar segments L3, L4, L5, and sacral medullary segment S1. This is because damage to the spinal conus can lead to urogenital, sensory, and/or motor dysfunction [4].

Spinal cord stimulation (SCS) is a technique that has been studied and appears to facilitate functional recovery by utilizing existing intact nerve connections within the spinal cord [5]. Here we present a case in which SCS was used to treat right lower extremity pain due to conus medullaris syndrome. This case highlights the diagnostic challenges inherent in conus medullaris syndrome and suggests the effectiveness of SCS in relieving neuropathic pain resulting from spinal cord injury. In conclusion, SCS therapy has proven to be an important treatment modality for patients suffering from conus medullaris syndrome, highlighting its important role in the management of chronic intractable neuropathic pain.

## Case presentation

**Figure 1:**
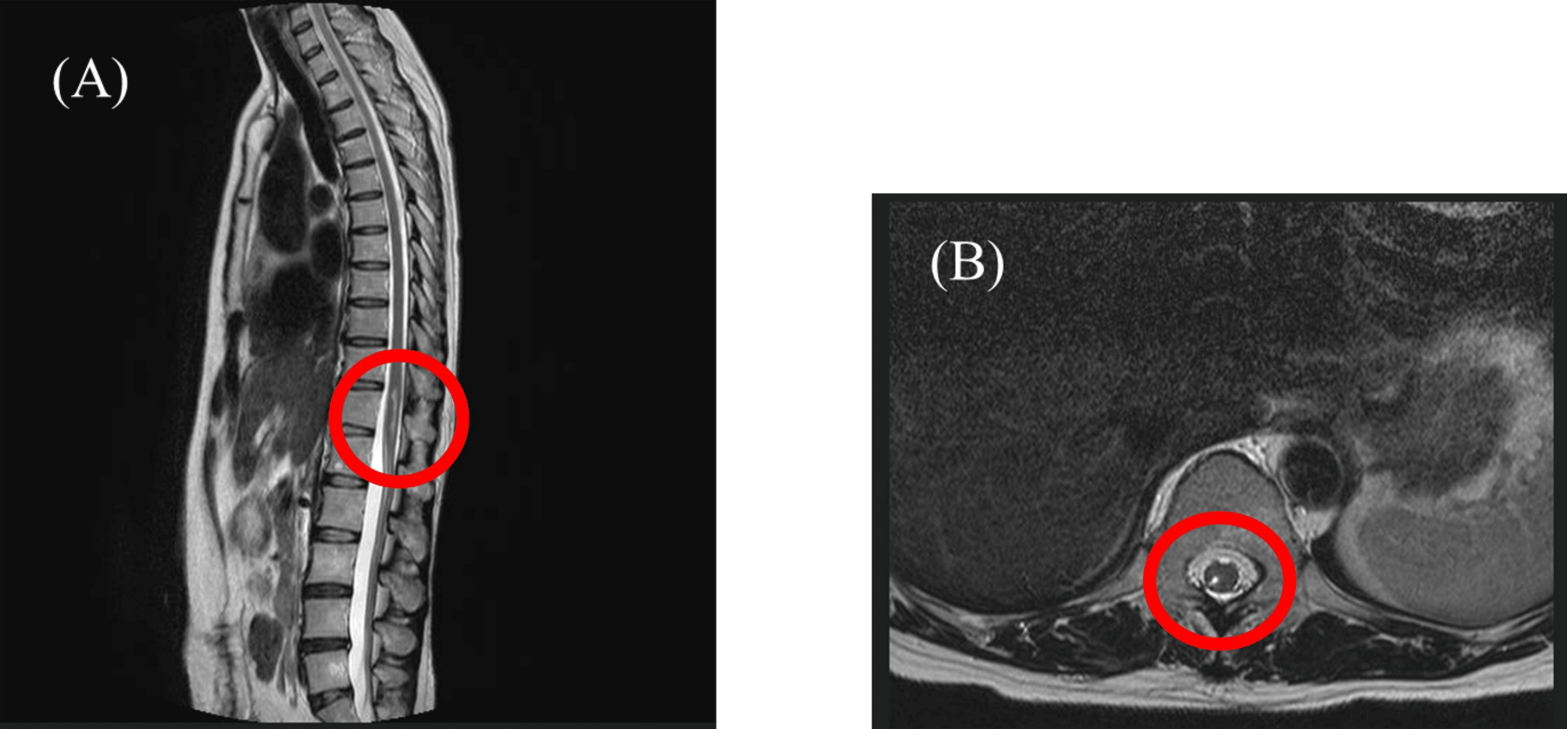
Edematous image of the spinal cord (right posterior horn to posterior cord) at the level of the 11th-12th thoracic vertebrae. No other lumbar spine disease was detected. (A) Sagittal plane; (B) transverse plane

A 49-year-old woman (151 cm, 53 kg, BMI 23.24) complained of right lower extremity pain and numbness. In December 2021, during epidural anesthesia for laparoscopic colon resection due to colon cancer, electric shock-like pain appeared in the right lower extremity. A catheter was placed for intraoperative and postoperative analgesia. After the epidural catheter was removed postoperatively, pain and numbness persisted throughout the right lower extremity. T2-weighted MRI images reflect tissue damage and increased water content due to edema as they extract water with high signal, and in this case, revealed damage to the thoracic and lumbar spinal cord with fluid in the region of the 12th thoracic cord. Because of persistent symptoms despite treatment, the patient was referred to our department in May 2022. There was no history other than a history of treatment for colorectal cancer and ovarian cysts. At the first visit, the patient reported pain from her right thigh to knee, with a Numeric Rating Scale (NRS) of 7/10 at rest and 10/10 during episodes. She walked with a limp, with pain being most severe during walking. Muscle weakness was noted in the right leg, and dorsiflexion of the ankle was occasionally difficult. MRI of the thoracolumbar spine showed edema at the T11-T12 spinal cord level (right posterior horn to posterior cord), with no other spinal pathologies or deformities (Figure 1).

**Figure 2:**
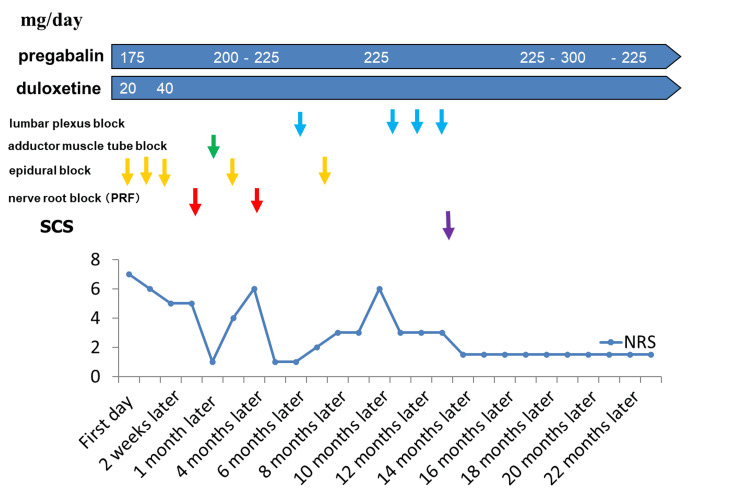
Course of treatment with nerve blocks and medications and course of NRS NRS: Numeric Rating Scale; PRF: pulsed radiofrequency; SCS: spinal cord stimulation blue arrows, lumbar plexus block; green arrow, adductor muscle tube block; yellow arrow, epidural block; red arrow, nerve root block; purple arrow, SCS

We suspected pain after spinal cord injury due to epidural anesthesia, but based on local symptoms (L3 nerve root distribution) from the right thigh to the knee, we thought it was nerve root pain. After epidural anesthesia and L3 nerve root block, pain was reduced from NRS 5 to 1. Numbness and toe discomfort persisted, but gait improved. However, the nerve root block was effective for only one to three months. The patient was started on pregabalin (175 mg) and duloxetine (40 mg) with moderate pain relief (NRS around 3). Because previous treatments had provided only temporary relief, SCS therapy (placement of leads at the superior border of T8 and T9, Abbott 40Hz/1000μs (Abbott Laboratories, Abbott Park, IL) was performed 14 months after the initial visit. This procedure significantly reduced the patient's pain and allowed her to engage in rehabilitation and resume his daily activities. This figure shows the treatment course of this case, showing that various nerve blocks were performed in addition to medication from the initial visit, but no lasting effect was obtained. After 14 months, SCS was implanted and the pain level stabilized at about NRS 3 (Figure 2).

## Discussion

In this case, the site of injury was at the level of the 11th-12th thoracic vertebrae, whereas the site of pain was at the level of the 2nd and 3rd lumbar spinal nerves, making the diagnosis difficult due to the pain discrepancy caused by traumatic spinal cord injury.

Considering that the spinal cord injury site was at the level of the 11th-12th thoracic vertebrae (right dorsal horn to posterior cord), the pain should have been contralateral, but in this case, the pain was ipsilateral. Symptomatically, there were no indications of a simple spinal cord injury. There was no deformity of the spinal column, including stenosis of other intervertebral foramina. However, it has been reported that when the injury site is located in the spinal conus (Th11 to L2 level), the concentration of segments from the L to S regions can result in conus medullaris syndrome with a variety of symptoms [2]. Conus medullaris syndrome is reported to occur near the upper (Th12) and lower (L1) ends of the spinal conus (L2), and when this area is affected, various symptoms are reported to appear [4]. Symptoms of conus medullaris syndrome are characterized by muscle weakness in the lower legs and below, muscle atrophy, and drooping legs, accompanied by vesico-rectal disturbances, radiculopathy, and sensory disturbance [6]. The pathology is reported to involve a mixture of upper and lower pyramidal neurons and may present with a variety of symptoms [7]. Although conus medullaris syndrome may not be accompanied by radiating pain, Toribatake et al. noted that the radiating distribution type often makes it difficult to differentiate from lumbar nerve root disorders, and reported that the radiating distribution type of conus medullaris syndrome should be kept in mind when examining patients with lower limb flaccid paraplegia disease [2]. Tokuhashi et al. also reported the site of the disorder and approximate symptoms of spinal supraspinal conus syndrome [8].

In the present case, the Th11-12 site was affected, with lower extremity pain and flaccid symptoms at the level of the spinal nerves that deviated from the site of the disorder. This is consistent with this report. Removal of the cause of the conus injury and steroid administration have been reported as treatment options for conus medullaris syndrome [9].

Spinal cord electrical stimulation therapy and rehabilitation have also been reported to be useful in spinal cord injury [5]. Regarding the analgesic mechanism of SCS therapy, stimulation of the posterior cord stimulates thick Aβ fibers, which retrogradely inhibits synaptic transmission at the level of the dorsal horn [10] and promotes the release of GABA and acetylcholine [11], resulting in the suppression of neuroexcitation in projection neurons and it is thought to exert its analgesic effect by inhibiting the neuronal excitability of projection neurons. An analgesic mechanism by activation of the serotonergic and noradrenergic descending inhibitory systems has also been reported [12]. SCS is considered effective for neuropathic and ischemic pain that is resistant to opioids and other drugs but is not effective for nociceptive pain. It is also contraindicated in cases of tumor invasion into the spine or spinal canal in the area of electrode placement. SCS is known to be highly effective for neuropathic pain among intractable pain [13] and was considered to be an appropriate indication for this patient who was refractory to conservative treatment. In fact, a sustained decrease in NRS was observed in this patient after SCS implantation. In addition, rehabilitation became possible and claudication gradually improved.

## Conclusions

This case highlights the diagnostic complexity of conus medullaris syndrome, where symptoms may mimic radicular pain. SCS therapy proved effective in treating lower limb pain that was resistant to conservative treatment. SCS should be considered for patients with neuropathic pain due to spinal cord injury when other treatments fail.
